# A Multi-Provincial Clinical Evaluation of the PANBIO™ COVID-19 Antigen Rapid Test Device in South Africa

**DOI:** 10.3390/diagnostics16142263

**Published:** 2026-07-20

**Authors:** Vidya Keshav, Lesley Scott, Anura David, Carey Pike, Linda-Gail Bekker, Wendy Stevens

**Affiliations:** 1 Wits Diagnostic Innovation Hub, Faculty of Health Sciences, University of the Witwatersrand, Johannesburg 2050, South Africa; 2 Desmond Tutu HIV Centre, University of Cape Town, Cape Town 7925, South Africa; 3 National Priority Program, National Health Laboratory Service, Johannesburg 2131, South Africa

**Keywords:** SARS-CoV-2, COVID-19, rapid antigen test, diagnostic performance, South Africa, point-of-care testing

## Abstract

**Background**: Rapid antigen tests were widely utilised during the COVID-19 pandemic to expand diagnostic access in resource-limited settings. However, prospective evaluations under intended-use conditions remained essential to define their role in decentralised testing strategies. **Methods**: We conducted a prospective multi-provincial clinical evaluation of the Panbio™ COVID-19 Antigen Rapid Test Device in South Africa between July 2021 and January 2022. Paired nasal and nasopharyngeal swabs were collected from 609 participants across three geographically diverse South African provinces. Antigen test results were compared with standard-of-care TaqPath RT-PCR, with BD MAX™ RT-PCR included as a secondary molecular comparator. Performance was assessed by viral load, symptom status, and variant predominance periods. **Results**: Standard-of-care RT-PCR identified 136 positive cases (22.3% prevalence). The Panbio™ test demonstrated overall sensitivity of 80.1% (95% CI: 72–87) and specificity of 98.5% (95% CI: 97–99). Sensitivity reached 90.1% in specimens with Ct values ≤25 and 85.2% among symptomatic participants tested within seven days of onset but declined at Ct values ≥30 and in asymptomatic individuals. Performance remained consistent during Delta- and Omicron-predominant periods. **Discussion**: The Panbio™ RDT demonstrates high diagnostic accuracy for detecting SARS-CoV-2 in individuals with high viral load, supporting its use for rapid identification of infectious cases during transmission surges. Molecular testing remains necessary for low viral load infections and asymptomatic screening.

## 1. Introduction

The COVID-19 pandemic highlighted critical gaps in global diagnostic capacity, particularly in low- and middle-income countries where laboratory infrastructure, reagent costs, and trained personnel limited access to molecular testing [1]. While reverse transcription polymerase chain reaction (RT-PCR) remains the reference standard for SARS-CoV-2 detection due to its high analytical sensitivity and specificity [2], its operational requirements, specialised laboratories, extended turnaround times, and supply chain dependencies limit its utility for rapid clinical decision-making and decentralised outbreak response [3].

Antigen-based rapid diagnostic tests (RDTs) emerged as a complementary strategy to address these limitations. By detecting viral nucleocapsid (N) proteins through lateral flow immunochromatography, these assays deliver results within 15–30 min at point-of-care, without requiring laboratory infrastructure [4,5]. This operational simplicity enabled large-scale testing during periods of high transmission and expanded access to communities where molecular testing was unavailable [6]. Although alternative point-of-care diagnostic approaches continue to emerge [7], antigen RDTs remained the most widely implemented decentralised testing technology during the COVID-19 pandemic [8]. Consequently, antigen RDTs became central to national testing strategies and were included in global procurement lists for epidemic preparedness [8,9].

However, the diagnostic performance of SARS-CoV-2 antigen RDTs has shown considerable variation across studies [10,11]. Reported sensitivities have ranged widely due to differences in study design, including the use of specimen types inconsistent with manufacturer instructions, evaluation of stored specimens, and inclusion of heterogeneous patient populations [11,12,13]. These inconsistencies emphasised the need for prospective evaluations conducted under intended-use conditions, with rigorous comparator testing and analysis of performance across viral load strata and clinical presentations [12,14].

The Panbio™ COVID-19 Antigen Rapid Test Device (Abbott Diagnostic GmbH, Jena, Germany) is a chromatographic immunoassay that detects SARS-CoV-2 N antigens in direct nasal or nasopharyngeal swabs using monoclonal antibodies targeting conserved epitopes of the N protein, with results interpreted visually within 15–20 min. The N protein is expressed in high copy numbers during viral replication and is highly conserved [15], making it a suitable target for antigen detection. Although previous studies have reported acceptable performance characteristics [16,17,18,19], most were conducted in high-income settings or used retrospective sample collections. Prospective data from geographically diverse, resource-limited settings with circulating variant strains remain limited.

South Africa experienced multiple SARS-CoV-2 waves driven by successive variants of concern, including Delta and Omicron, placing sustained pressure on diagnostic systems [20]. The National Health Laboratory Service in South Africa processed millions of RT-PCR tests [21] during the pandemic, yet decentralised testing options remained essential for community-level case identification and cross-border testing [22]. Evaluating antigen RDT performance in this context across provinces, variant periods, and symptomatic presentations was necessary to define their appropriate role in the public health response.

We therefore conducted a prospective multi-provincial clinical evaluation of the Panbio™ COVID-19 Antigen Rapid Test Device in South Africa during periods of high SARS-CoV-2 transmission. This study aimed to determine diagnostic accuracy compared with standard-of-care (SOC) RT-PCR, assess performance across viral load categories and symptom duration, and evaluate consistency during Delta- and Omicron-predominant periods using a secondary molecular comparator.

## 2. Materials and Methods

### 2.1. Study Design and Ethics

We conducted a prospective diagnostic accuracy study at three clinical sites in South Africa, located in KwaZulu-Natal (Durban), Western Cape (Cape Town), and Gauteng (Johannesburg), selected to represent geographically diverse participant populations, between July 2021 and January 2022. The study was registered with the South African National Clinical Trials Registry (Trial ID: 5559) and received ethical approval from the Human Research Ethics Committee (Medical) of the University of the Witwatersrand (Reference no: M200468) and the University of Cape Town (Reference no: 732/2020). Regulatory approval was obtained from the South African Health Products Regulatory Authority (MD20200701). All participants provided written informed consent prior to enrolment.

### 2.2. Participant Enrolment

A total of 611 participants were enrolled consecutively from individuals presenting at participating clinical sites. Eligible participants were adults aged 18 years or older who met at least one of the following criteria: (i) self-reporting symptoms consistent with SARS-CoV-2 infection (fever, sore throat, cough, headache, anosmia, runny nose, body aches, fatigue, or diarrhoea); (ii) reporting close contact with a laboratory-confirmed COVID-19 case within the previous seven days; or (iii) reporting recent travel to areas with high SARS-CoV-2 transmission within the past 30 days. To ensure a representative adult population, enrolment of participants aged 65 years or older was limited to 9% of the total cohort. There was no exclusion criteria related to pregnancy, HIV status, or comorbidities.

### 2.3. Specimen Collection

For each participant, trained study staff collected three respiratory specimens in a standardised sequence. First, an anterior nasal swab was collected for Panbio™ antigen testing by rotating the swab against the nasal wall in both nostrils according to the manufacturer’s instructions. This swab was immediately inserted into the provided extraction buffer for on-site testing. Second, two nasopharyngeal (NP) swabs, one from each nostril, were collected for RT-PCR testing. The first NP swab was transported to the local provincial reference laboratory for SOC RT-PCR testing (within 24 h). The second NP swab was placed into 3 mL of universal transport medium (UTM, Copan Diagnostics, Murrieta, CA, USA) and transported at 2–8 °C to a central research laboratory in Johannesburg for batch molecular testing using a secondary comparator assay.

### 2.4. Panbio^TM^ RDT Procedure

Testing was performed at the collection site by trained study staff following the manufacturer’s instructions. Briefly, the nasal swab was eluted in extraction buffer, and five drops of the extracted specimen were added to the test cassette’s sample well. Results were visually read between 15–20 min; readings after 20 min were considered invalid. A visible control line and test line indicated a positive result; a visible control line only indicated a negative result; absence of the control line indicated an invalid test, which was repeated with a new device. To ensure quality assurance review and future verification, digital photographs of all test cassettes were captured and stored in a secure electronic database.

### 2.5. Reference Standard RT-PCR Testing

Provincial reference laboratories performed SOC RT-PCR testing using the TaqPath™ COVID-19 CE-IVD RT-PCR Kit on the QuantStudio™ 5 or 7 Real-Time PCR System (Thermo Fisher Scientific, Waltham, MA, USA). This assay targets three SARS-CoV-2 gene regions: open reading frame 1ab (*ORF1ab*), nucleocapsid (*N*), and spike (*S*). Amplification conditions were applied according to the manufacturer’s protocol, with results interpreted using the associated software. A specimen was considered positive if at least two of the three targets amplified with a cycle threshold (Ct) value of ≤36. Ct values for each target gene were recorded for all positive specimens. For viral load stratification, the *N* gene Ct values were used as a direct comparable measure to the N protein detecting Panbio™ RDT.

### 2.6. Secondary Comparator RT-PCR

Specimens collected in UTM were transported to the WITS Diagnostic Innovation Hub research laboratory in Johannesburg and stored at −80 °C until batch testing. Following a single freeze–thaw cycle, specimens were tested using the BD MAX™ SARS-CoV-2 assay on the BD MAX™ System (Becton Dickinson, Franklin Lakes, NJ, USA). This assay targets two conserved regions of the nucleocapsid gene (*N1* and *N2*) and includes *RNAseP* as an internal control for specimen adequacy. Results were interpreted using the manufacturer’s algorithms, with Ct values recorded for the N1 and N2 targets.

### 2.7. Data Management

Demographic information, clinical symptoms, symptom onset dates, exposure history, and test results were recorded on case report forms and entered into a secure electronic database (REDCap, Vanderbilt University, Nashville, TN, USA; hosted at the University of Cape Town, South Africa). Data entered was confirmed by independent operators and validated against source documents. All patient-identifying information was removed prior to analysis. Data management procedures were implemented to maintain data integrity and ensure independent analysis. Academic investigators retained control over study design, data collection, and statistical analysis, with no sponsor involvement in these activities or access to study data prior to completion of the analysis. Data validation was performed by an independent clinical partner (Epicentre), and the sponsor was informed of the final study results only after completion of the analysis.

### 2.8. Statistical Analysis

Data analysis was performed using STATA version 14 (StataCorp, College Station, TX, USA). The diagnostic accuracy of the Panbio™ RDT was evaluated against the SOC TaqPath RT-PCR result as the primary reference standard. Sensitivity, specificity, positive predictive value (PPV), and negative predictive value (NPV) were calculated with 95% confidence intervals (CIs). Agreement between the RDT and RT-PCR was evaluated using Cohen’s kappa coefficient (κ), with values interpreted as: ≤0.20 as poor, 0.21–0.40 as fair, 0.41–0.60 as moderate, 0.61–0.80 as good, and 0.81–1.00 as very good agreement.

## 3. Results

### 3.1. Study Population

Of the 611 participants enrolled, 2 were excluded from the final analysis: 1 developed epistaxis after nasal swab collection, and 1 withdrew consent during specimen collection. The final analysis included 609 participants. Participant ages ranged from 20 to 78 years, with a median age of 40 years (SD ± 13.19). Among the 609 participants, 525 (86.21%) self-reported symptoms consistent with SARS-CoV-2 infection (≤7 days), 81 (13.30%) reported recent exposure to confirmed cases (≤7 days), and 3 (0.5%) reported recent travel to high transmission areas (≤30 days). Participant characteristics are summarised in Table 1.

### 3.2. Diagnostic Performance of the Panbio^TM^ RDT

The SOC Taqpath RT-PCR assay identified 136 positive cases among the 609 participants, corresponding to a prevalence of 22.3% in the study population. The Panbio™ RDT detected 109 (80.14%) true positive cases and 466 (98.52%) true negative cases. Performance results are summarised in Table 2.

**Figure 1:**
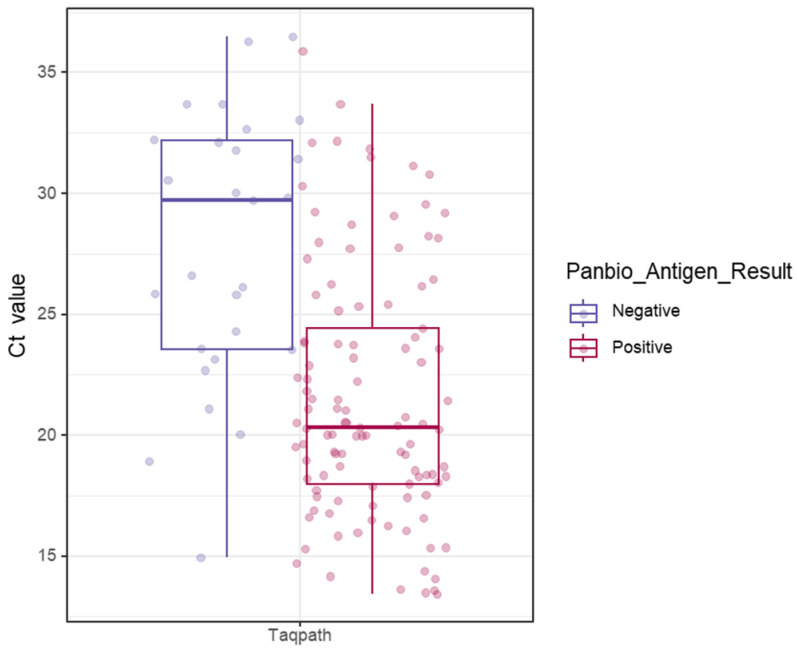
Boxplots show the distribution of *N* gene Ct values for SARS-CoV-2 detection using the TaqPath RT-PCR platform, categorised by Panbio^TM^ antigen test results (positive: red; negative: blue).

Diagnostic sensitivity of the Panbio™ RDT showed strong association with viral load as reflected by *N* gene Ct values from the TaqPath assay. For specimens with high viral loads (HVLs, Ct ≤ 25, *n* = 91), the Panbio™ RDT detected 82, yielding a sensitivity of 90.1% (95% CI: 82–95). For specimens with medium viral loads (MVL, Ct 25–30, *n* = 24), the Panbio™ RDT detected 18, yielding a sensitivity of 75% (95% CI: 53–90). For specimens with low viral loads (LVL, Ct ≥ 30, *n* = 21), the Panbio™ RDT detected only 9, yielding a sensitivity of 42.9% (95% CI: 22–66). Figure 1 illustrates the distribution of Ct values for Panbio^TM^-positive and Panbio^TM^-negative specimens.

### 3.3. Performance Compared with BD MAX^TM^ RT-PCR Assay

Of the 609 specimens intended for analysis with the BD MAX™ comparator RT-PCR assay, 5 specimens leaked during transportation. Consequently, 604 specimens were analysed. The Panbio^TM^ RDT showed an overall sensitivity of 72.4% (95% CI: 65–79) and a specificity of 99.6% (95% CI: 98–100) compared to the BD MAX™ assay. Despite limitations such as specimen dilution in 3 mL of universal transport medium and delayed batch testing, the BD MAX™ RT-PCR detected 36 additional positive specimens that were not identified by the TaqPath SOC assay. Among these discordant positives, five cases were also identified by the Panbio™ antigen test with Ct values ranging from 17.8–26.1 for the *N1* gene. The higher detection rate of the BD MAX™ assay likely reflects its dual N1/ N2 target design, whereas the TaqPath assay includes the *S* gene, which is more prone to mutation-related target failure. These results indicate that discordances between the Panbio™ RDT and the Taqpath RT-PCR reference assay may reflect differences in molecular assay sensitivity rather than erroneous RDT results.

### 3.4. Performance According to Symptom Status and Duration

**Figure 2:**
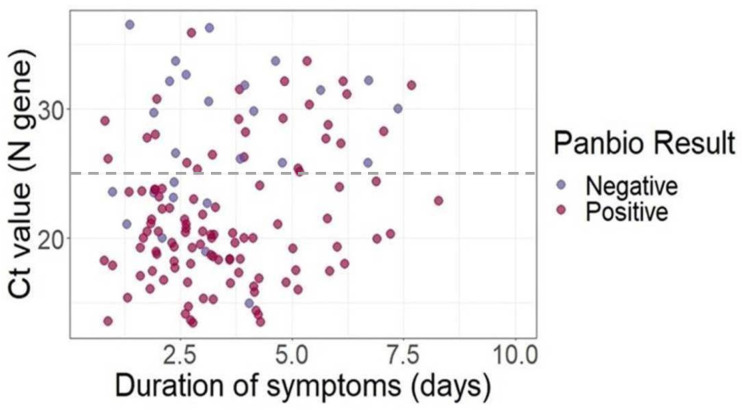
Relationship between SARS-CoV-2 RT-PCR cycle threshold (Ct) values and symptom duration (days), grouped by Panbio^TM^ rapid antigen test results (positive: red; negative: blue). The dashed horizontal line indicates a Ct value threshold of 25.

Among 525 symptomatic participants, Taqpath RT-PCR detected SARS-CoV-2 in 135 individuals (25.7%). The Panbio™ RDT correctly identified 115 (85.2%) of these 135 symptomatic positive cases. Among the 84 asymptomatic participants, Taqpath RT-PCR detected 1 positive case (1.2%), which was also detected by the Panbio™ RDT. Sensitivity varied with symptom duration at the time of testing. Among symptomatic participants with Taqpath RT-PCR confirmed infection, the highest detection rates were observed on days 2 and 3 following symptom onset. Figure 2 illustrates the relationship between symptom duration, Ct values, and Panbio™ RDT positivity. To further evaluate the influence of viral load on test performance across the symptomatic period, diagnostic sensitivity was stratified by symptom duration and viral load category (Supplementary Table S1). Sensitivity remained consistently high for HVL specimens (Ct ≤25) across all symptom duration categories, whereas sensitivity declined substantially for LVL specimens (Ct >30). These results indicate that viral load, rather than symptom duration alone, was the primary determinant of Panbio™ RDT performance.

### 3.5. Performance During Circulation of Different SARS-CoV-2 Variants

**Figure 3:**
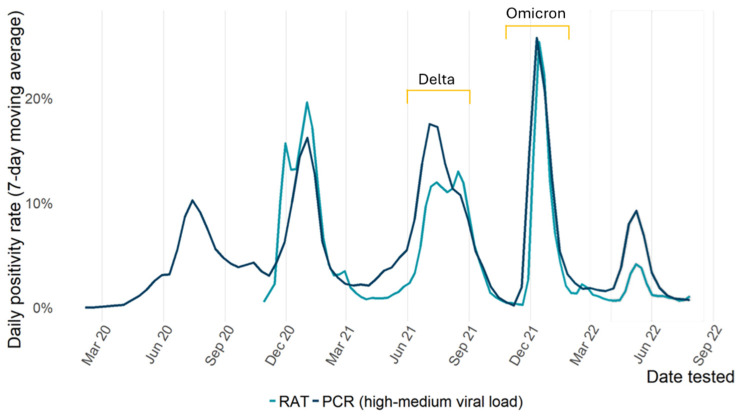
National Health Laboratory Service’s continuous quality monitoring of RT-PCR (Ct ≤ 30) and rapid antigen tests (RATs) positivity rates during SARS-CoV-2 infection waves in South Africa, with the study period aligning with the dominance of the Delta and Omicron variants.

Analysis of enrolment dates relative to national surveillance data showed that 580 participants (95.2%) were enrolled during the Delta variant predominance period (July to November 2021), and 29 participants (4.8%) during the early Omicron wave (December 2021 to January 2022). During the Delta predominant period, the Panbio™ RDT demonstrated a sensitivity of 79% (95% CI: 71–86) and specificity of 98.7% (97–100). During the Omicron predominant period, sensitivity was 92% (95% CI: 62–100) and specificity was 94% (95% CI: 71–100). Viral sequencing was not performed. Variant attribution was inferred from temporal trends in national surveillance data (Figure 3).

## 4. Discussion

This prospective multi-provincial evaluation showed that the Panbio™ RDT achieved an overall sensitivity of 80.1% and specificity of 98.5% when compared with SOC RT-PCR in South Africa. These results align with previous evaluations [19,23,24] conducted in high-transmission settings and support the integration of antigen RDTs into decentralised testing strategies, especially for symptomatic individuals during periods of high transmission.

The strong association between diagnostic sensitivity and viral load observed in this study aligns with the known limitations of lateral flow immunoassays [25]. Sensitivity exceeded 90% in specimens with Ct values ≤25, which correspond to HVL and a higher likelihood of infectiousness [17,26]. This has important public health implications; the Panbio™ RDT reliably detects individuals most likely to be actively transmitting SARS-CoV-2, allowing rapid isolation and contact tracing. Conversely, sensitivity declined to 42.9% in specimens with Ct values ≥30, indicating that antigen testing is unsuitable for ruling out infection in individuals with LVL [25]. These results reinforce WHO guidance advising against routine RDT use for asymptomatic screening outside high-risk settings [8,26] and emphasise the ongoing need for molecular testing when clinical suspicion persists despite a negative antigen result.

The temporal pattern of detection observed in this study, with peak sensitivity on days 2–3 following symptom onset, reflects the natural course of SARS-CoV-2 viral kinetics [27]. Peak viral loads typically occur around the time of symptom onset or shortly thereafter, with infectious virus shedding declining after day 5–7 [28,29,30]. These dynamics explain the lower sensitivity observed in our study among participants tested more than 7 days after symptom onset and support current recommendations that antigen testing should be prioritised early in the course of illness. Testing algorithms that consider symptom duration may optimise RDT yield and reduce false-negative results [13].

An important methodological contribution of this study was the inclusion of a secondary molecular comparator, the BD MAX™ RT-PCR assay. This identified 36 additional positive specimens not detected by the TaqPath SOC assay. Five of these discordant specimens were also detected by the Panbio™ RDT, with N1 Ct values ranging from 17.8–26.1, consistent with HVL infections. Differences in analytical sensitivity, extraction protocols, and target design may have contributed to the discordance observed between the molecular assays. The BD MAX™ assay targets two conserved regions of the nucleocapsid gene (*N1* and *N2*), whereas the TaqPath™ assay includes the S gene, which may be susceptible to mutation-related target failure during periods of viral evolution [31,32,33,34].

These findings suggest that some apparent Panbio™ false-positive results may instead represent true infections not detected by the primary molecular comparator. They also highlight the importance of using molecular reference assays that incorporate multiple conserved targets when evaluating diagnostic performance. Molecular assays targeting highly conserved regions, such as the *E* gene, may provide additional resilience against mutation-related target failure [35].

Despite concerns about mutations in the N protein of recent variants [36,37], the Panbio™ RDT maintained consistent performance during both Delta- and Omicron-predominant periods in this study. Sensitivity during the Omicron period (92%) was comparable to that observed during Delta (79%), although confidence intervals overlapped considerably due to limited sample size during the Omicron wave. These data are reassuring and align with other reports indicating that commercially available RDTs have largely retained their detection capability across variants of concern, despite theoretical risks of immune evasion at the N epitope level [34]. Continuous post-market surveillance is necessary as new variants emerge, and viral sequencing should be incorporated into future evaluations where it is feasible to confirm variant attribution.

The operational advantages of antigen RDTs merit emphasis in the context of health systems with constrained laboratory capacity. The Panbio™ test provides results within 15–20 min at the point of care, supporting immediate clinical triage, isolation decisions, and initiation of contact tracing interventions that cannot await laboratory RT-PCR results [3,5]. During pandemic surges, when laboratory systems become overwhelmed, RDTs offer an adaptable strategy for maintaining testing access at the community level [38,39]. However, these operational benefits must be balanced against the diagnostic trade-offs identified in this study, including reduced sensitivity for LVL infections and for asymptomatic individuals. Testing strategies should therefore align with intended use. Antigen RDTs are most suitable for rapid identification of infectious individuals during periods of high transmission, whereas molecular testing remains important for high-risk patients, confirmation of clinically suspicious negative RDT results, and asymptomatic screening programmes.

Real-world implementation of antigen RDTs within South African health systems also requires consideration of operational barriers, including user training, quality assurance for visual interpretation of faint test lines, supply chain reliability, and waste management at decentralised testing sites. These factors may influence the scalability and sustainability of RDT-based testing strategies and should be incorporated into implementation planning.

This study has several limitations. First, the asymptomatic cohort was small (*n* = 84), which limits the accuracy of estimating antigen test performance for asymptomatic screening. Asymptomatic and pre-symptomatic transmission are important factors in SARS-CoV-2 dynamics, and larger studies specifically enrolling asymptomatic individuals are needed to define RDT performance in this population. Second, the sample size during the Omicron-predominant period was limited, and variant attribution was based on national surveillance data rather than individual viral sequencing. Subsequent evaluations should include sequencing to confirm variant-specific performance. Third, visual interpretation of faint test bands may introduce operator variability, particularly in low-prevalence settings where positive results are infrequently encountered [40,41,42]. Digital RDT readers or smartphone-based applications incorporating artificial intelligence could improve result standardisation and reporting accuracy in large-scale screening [40,43], and should be considered for future implementation. Fourth, the BD MAX™ comparator testing was performed on specimens diluted in 3 mL of universal transport medium and subjected to freeze–thaw cycles during batch testing, which may have affected RNA integrity and detection levels. However, the higher positivity rate observed with BD MAX™ compared to TaqPath suggests that any degradation was insufficient to mask the assay’s increased analytical sensitivity. Fifth, although the N protein contains conserved motifs shared across the Coronaviridae family that could theoretically contribute to cross-reactivity, the high specificity observed in this study (98.5%) suggests that clinically significant cross-reactivity was minimal. Lastly, this study was conducted during periods of Delta and Omicron predominance; performance against future variants cannot be assumed and requires continuous assessment.

## 5. Conclusions

The Panbio™ COVID-19 Antigen Rapid Test Device demonstrates high diagnostic accuracy for detecting SARS-CoV-2 in individuals with HVLs, particularly when tested within the first three days of symptom onset. This supports its use as a public health tool for rapid identification of infectious cases during transmission surges, where its operational advantages can be fully realised. Molecular testing remains essential for detecting LVL infections, screening asymptomatic populations, and confirming negative RDT results when clinical suspicion persists. Performance in asymptomatic individuals could not be robustly assessed because only one asymptomatic SARS-CoV-2-positive participant was identified. Further studies are required before routine use in asymptomatic screening programmes can be recommended. As outbreak preparedness remains a global priority, antigen RDTs will remain an important part of the diagnostic landscape, provided their performance is continuously monitored, and their use aligns with clearly defined testing objectives.

## Figures and Tables

**Table 1:** Demographic and Clinical Characteristics of Study Participants.

Total Number of Participants	609
Location	142 Durban (23%), 376 Cape Town (62%), 91 Johannesburg (15%)
Gender	274 Male (45%), 335 Female (55%)
Age (years)	127: 18–25 (21%), 244: 26–40 (40%), 193: 41–60 (32%), 45: >60 (7%)
Symptomatic profile	84 asymptomatic (14%), 525 symptomatic < 7 days (86%)
cough	54% (331)
runny nose	51% (309)
sore throat	52% (314)
Fever	29% (176)
Headache	56% (341)
Body aches	39% (236)
Diarrhoea	11% (68)
Vomiting/nausea	15% (89)
Anosmia	14% (86)
Other *	9% (56)

* Chills, heartburn, fatigue, vertigo, congestion, sore eyes, blocked nose, foggy head, itchy eyes, shortness of breath, sneezing, dyspnoea, loss of appetite, sweat.

**Table 2:** Diagnostic Performance of the Panbio^TM^ RDT Compared with Taqpath RT-PCR (*N* = 609).

Characteristics	Performance (95% CI)
Overall Sensitivity	80% (72–87)
Overall Specificity	98.5% (97–99)
Positive Predictive Value	94% (88–98)
Negative Predictive Value	94.5% (92–96)
Cohen’s kappa (k)	0.83 (0.78–0.89)
Level of Agreement	Very good

## Data Availability

All relevant data are presented within the manuscript. No publicly archived datasets were generated or analysed during this study.

## Supplementary Materials

The following supporting information can be downloaded at: https://www.mdpi.com/article/10.3390/diagnostics16142263/s1, Table S1: Diagnostic Sensitivity of the Panbio™ RDT Stratified by Days Since Symptom Onset and Viral Load Category.
