# Supplementary material for: A Multi-Provincial Clinical Evaluation of the PANBIO™ COVID-19 Antigen Rapid Test Device in South Africa
Source: Diagnostics (Basel). 2026 Jul 20;16(14):2263. doi: 10.3390/diagnostics16142263 (PMC13409381; doi:10.3390/diagnostics16142263)
Supplement: Supplementary file 1 [file diagnostics-16-02263-s001.zip › diagnostics-4327361-supplementary.pdf]

diagnostics

Supplementary Material – Cross-stratified analysis

Supplementary Table S1. Diagnostic Sensitivity of the Panbio™ RDT Stratified by Days Since Symptom Onset and Viral Load Category.

Sensitivity estimates with 95% confidence intervals are presented according to days since symptom onset and SARS-CoV-2 viral load category. Viral load stratification was based on TaqPath™ N gene cycle threshold (Ct) values and defined as high viral load (HVL, Ct ≤25), medium viral load (MVL, Ct 25-30), and low viral load (LVL, Ct >30). This cross-stratified analysis was performed to evaluate the combined influence of symptom duration and viral load on Panbio™ RDT performance. Sensitivity remained highest among high viral load specimens across all symptom duration categories, whereas lower sensitivity was generally observed among low viral load specimens. Cells where no positive cases were detected by the Panbio™ RDT are indicated accordingly.

Day of Symptom Onset	Viral Load Category	Panbio™ RDT Positive (n)	TaqPath RT-PCR Positive (n)	Sensitivity (95% CI)	Specificity (95% CI)
Day 1	HVL (Ct <25)	21	25	84% (64-96)	98% (93-100)
	MVL (Ct 25-30)	5	6	83% (36-100)	98% (93-100)
	LVL (Ct >30)	0	2	0% (0-84)	98% (93-100)
Day 2	HVL (Ct <25)	28	32	88% (71-97)	97% (92-99)
	MVL (Ct 25-30)	3	5	60% (15-95)	97% (92-99)
	LVL (Ct >30)	3	7	43% (10-82)	97% (92-99)
Day 3	HVL (Ct <25)	15	15	100% (78-100)	100% (95-100)
	MVL (Ct 25-30)	2	2	100% (16-100)	100% (95-100)
	LVL (Ct >30)	2	4	50% (7-93)	100% (95-100)
Day 4	HVL (Ct <25)	12	13	92% (64-100)	100% (92-100)
	MVL (Ct 25-30)	3	5	60% (15-95)	100% (92-100)
	LVL (Ct >30)	2	4	50% (7-93)	100% (92-100)
Day 5	HVL (Ct <25)	3	3	100% (29-100)	100% (87-100)
	MVL (Ct 25-30)	3	3	100% (29-100)	100% (87-100)
	LVL (Ct >30)	2	2	100% (16-100)	100% (87-100)
Day 6	HVL (Ct <25)	1	1	100% (3-100)	83% (40-100)
	MVL (Ct 25-30)	1	1	100% (3-100)	83% (40-100)
	LVL (Ct >30)	0	0	0% (No positives detected on Panbio™)	
Day 7	HVL (Ct <25)	2	2	100% (16-100)	100% (74-100)
	MVL (Ct 25-30)	0	1	0% (No positives detected on Panbio™)	
	LVL (Ct >30)	0	1	0% (No positives detected on Panbio™)	

Abbreviations: HVL, high viral load; MVL, medium viral load; LVL, low viral load; Ct, cycle threshold; CI, confidence interval; RDT, rapid diagnostic test; Panbio™, Panbio™ COVID-19 Antigen Rapid Test Device (Abbott Diagnostic GmbH, Jena, Germany); Taqpath, TaqPath™ COVID-19 CE-IVD RT-PCR Kit on the QuantStudio™ 5 or 7 Real-Time PCR System (Thermo Fisher Scientific, USA).
